# QSSI (QGIS summer simmer index) calculator plugin: an open-source tool for thermal comfort analysis in gis applications

**DOI:** 10.1007/s00484-026-03180-x

**Published:** 2026-03-24

**Authors:** Fatih Adiguzel, Mansur Bestas, Enes Karadeniz, Asir Yuksel Kaya, Rukiye Gizem Oztas Karli

**Affiliations:** 1 Department of Transportation Services, Technical Sciences Vocational School, Bitlis Eren University, Bitlis, 13000 Türkiye; 2 Bitlis Eren University, Faculty of Economics and Administrative Sciences, Business Department, Bitlis, 13000 Türkiye; 3 Department of Geography, Faculty of Science and Letters, Inonu University, Malatya, Türkiye; 4 Department of Geography, Faculty of Humanities and Social Sciences, Fırat University, Elazığ, 23000 Türkiye; 5 Faculty of Engineering, Architecture and Design, Department of Landscape Architecture, Bartin University, Bartin, Türkiye

**Keywords:** Thermal Comfort, Summer Simmer Index, Adana, GIS, QSSI

## Abstract

Thermal stress, driven by the combined influence of high temperature and humidity, has become a critical public health concern in rapidly urbanizing Mediterranean cities. This study introduces the QGIS Summer Simmer Index (QSSI) Calculator, an open source plugin developed in Python and Qt Designer to provide fast and reproducible mapping of summer thermal stress. The tool integrates the Summer Simmer Index (SSI) directly into the QGIS environment, enabling users to generate spatially explicit bioclimatic stress maps without relying on external modeling platforms. Adana was selected as the pilot area due to its prolonged hot and humid summer conditions. The results indicate that more than 80% of the urban population is exposed to Hot or Very Hot SSI classes during the summer season, highlighting significant risks for nearly 1.8 million residents. These findings demonstrate the plugin’s value as a practical spatial decision support tool for climate adaptation and urban heat management. Future versions will incorporate additional environmental variables to improve model detail and broaden applicability.

**Supplementary Information:**

The online version contains supplementary material available at 10.1007/s00484-026-03180-x.

## Introduction

The rapid increase in urbanization today has brought new challenges. These issues, mostly related to climatic factors, include the greenhouse gas effect, the urban heat island effect, increased energy demand, and the decline in urban life quality (Bai et al. 2017; Das et al. 2024; Ren et al. 2022; Santamouris 2014; Santamouris et al. 2015). One of these challenges, the disruption of the thermal comfort balance, is considered a more prominent issue in urban planning as it directly affects public health and quality of life. This problem, which becomes more hazardous with rising temperatures, limits individuals’ capacity to adapt to environmental conditions (Cetin et al. 2024; Kawamoto 2017; Marchesi et al. 2014; Schlegel et al. 2020; Singh et al. 2020; Vanos et al. 2010). Therefore, ensuring thermal comfort has become a necessity, particularly in regions with high urbanization rates (Cetin 2019; Çağlak 2024; Olgyay 2016; Santamouris et al. 2015).

While the concept of thermal comfort was scientifically modeled by Fanger (1970), Olgyay (1973) emphasized its importance in the context of bioclimatic architectural design, highlighting the role of climate in shaping thermally comfortable environments. Thermal comfort aims to measure the extent to which individuals feel comfortable and healthy under specific climatic conditions by analyzing the combined effects of variables such as temperature, humidity, wind speed, and solar radiation (Adiguzel et al. 2020; Cetin 2019; Cetin et al. 2019, 2023; Marn et al. 2019).

The Universal Thermal Climate Index (UTCI), Physiological Equivalent Temperature (PET), WindChill Index, and NEN 8100, which are used to evaluate thermal comfort in different climate zones, are commonly preferred in outdoor space analyses (Höppe 1999a, b; Korobeinikova et al. 2024; Matzarakis et al. 1999; Mayer and Höppe 1987; Tseliou et al. 2010). These indices comprehensively analyze human thermal perception by combining numerous meteorological and physiological parameters, including air temperature, humidity, wind speed, radiation fluxes, clothing insulation, and metabolic rate (Höppe 1999a, b; Matzarakis et al. 1999; Jendritzky et al. 2012; Bröde et al. 2012). While these indices offer high physiological accuracy, they also have certain limitations. Their application often requires assumptions about multiple meteorological variables, as well as parameters such as human activity and clothing insulation, and frequently relies on specialized or external modeling environments. This situation can limit their usability in large-scale or data-constrained spatial analyses (Bröde et al. 2012; Matzarakis et al. 2007).

The Summer Heat Index (SSI) differs from other indices in that it specifically focuses on thermal stress caused by the combined effects of air temperature and relative humidity during the summer months (Arıcak 2020; Sancar and Güngör 2020; Zhu et al. 2019). Compared to many other bioclimatic indices, the SSI offers a relatively simple calculation approach that assesses perceived heat stress under hot and humid conditions based solely on two commonly available input variables: air temperature and relative humidity (Pepi 1987a, b; Steadman 1979). SSI is particularly suitable for the Mediterranean climate, characterized by prolonged summer heat waves and high humidity levels. This is because humidity in these regions significantly increases thermal discomfort. From this perspective, SSI does not aim to replace comprehensive thermal comfort indices such as UTCI or PET. Instead, it positions itself as a complementary tool to these indices by offering a practical and operationally efficient option for spatial analyses where data availability, ease of calculation, and interpretability are critical.

Thermal comfort analyses play a crucial role in urban planning. These analyses offer various benefits, including the consideration of climatic characteristics in urban planning, the mitigation of the urban heat island effect, and the enhancement of individuals’ adaptation to environmental conditions (Battisti 2020; Cetin et al. 2019; Çağlak 2024; Gungor et al. 2021; Korobeinikova et al. 2024; Vanos et al. 2010). However, the significance of thermal comfort in urban planning is not limited to improving individuals’ daily quality of life. The use of thermal comfort analyses as a tool in the formulation of urban policies also serves as a fundamental strategy for ensuring that urban areas remain sustainable and livable in the long term.

Thermal comfort analyses can be conducted using various software programs and tools. QGIS, an open-source and user-friendly GIS software, is widely used in various fields (Duarte and Teodoro 2021; Graser and Olaya 2015; Jaya and Fajar 2019) (QGIS Development Team, 2024). Additionally, QGIS provides the capability to develop custom plugins for spatial analysis needs (Correia et al. 2018; Duarte and Teodoro 2021; Foglia et al. 2018; Rosas-Chavoya et al. 2022). This capability has led to the development of numerous plugins for applications in water management, land use, remote sensing, geology, archaeology, risk analysis, urban planning, and library sciences (Becker et al. 2016; Chen et al. 2024; Guo et al. 2022; Locati et al. 2021; López-Ballesteros et al. 2024; Naciri et al. 2024; Renard et al. 2024; Rossetto et al. 2022; Scala et al. 2024; Tobias and Mandel 2021; Verma et al. 2024).

Within this ecosystem, various QGIS-based tools and workflows have been developed to support microclimatic, bioclimatic, and environmental thermal analyses (Isinkaralar 2024; Sola-Caraballo et al. 2025). Current approaches typically focus on radiation modeling, mean radiant temperature estimation, urban heat island assessment, or generalized climate mapping (Touati et al. 2020). RayMan-based workflows and radiation-focused plugins such as SOLWEIG enable the calculation of mean radiant temperature and related thermal comfort indicators by representing short- and long-wave radiation processes in detail, thereby providing detailed assessments of the radiation component of microclimate. However, the application of these tools often relies on detailed pre-processing steps, multiple meteorological inputs, and technical knowledge of the relevant physical processes (Lindberg et al. 2018; Matzarakis et al. 2007; Thorsson et al. 2007). This can limit the direct applicability of these tools in urban planning applications.

From a methodological perspective, existing GIS-based studies addressing thermal comfort and bioclimatic conditions can generally be divided into three main approaches: radiation and microclimate-focused modeling (Colaninno et al. 2025; Wallenberg et al. 2022), generalized climate or urban heat island mapping (Touati et al., 2020; Sola-Caraballo et al. 2025), and the integration of comprehensive bioclimatic indices such as PET or UTCI through complex modeling environments (Çağlak and Türkeş 2023; Nath and Deka 2025). From a climate adaptation perspective, recent GIS-based studies increasingly treat thermal comfort analysis as a decision support mechanism for urban heat resilience and climate adaptation planning (Cho et al. 2025; Ravnikar et al. 2025). Current research focuses on pedestrian-scale heat exposure mapping, the integration of thermal indicators with urban morphology and green infrastructure, and the evaluation of adaptation scenarios under current and future climate conditions (de Quadros and Mizgier 2023; Sola-Caraballo et al. 2025; Isinkaralar 2024; Cho et al. 2025; Isinkaralar et al. 2025). However, simple and easy to use GIS applications designed specifically to reveal summer heat stress remain limited. Despite this comprehensive ecosystem of spatial analysis tools, a user-focused, specialized QGIS plugin explicitly focused on the spatial analysis of summer thermal stress through the SSI framework has not been made available to date. To address this gap, a Summer Heat Index (SSI) plugin has been developed and integrated into the QGIS software.

This study aims to introduce the “SSI CALCULATOR” plugin, which has been integrated into QGIS to analyze the effects of summer temperatures in a detailed and spatial manner. In line with this objective, the applicability and usability of this plugin are tested in the case of Adana. The selection of Adana as the study area is influenced by several factors: its exposure to high temperatures and humidity levels during the summer months due to the effects of the Mediterranean climate (MGM 2024), the frequent occurrence of thermal stress and thermal comfort losses (Cetin et al. 2016; Adiguzel et al. 2022; Adıgüzel and Doğan 2021; Çağlak 2024), and its high urban density (Kalli et al. 2019; Cilek and Cilek 2021; Ünal 2022; Yelekçi 2019). Adana’s environmental conditions align well with the characteristics of the Summer Simmer Index. For this reason, selecting SSI as the primary indicator in this study is appropriate both in terms of the climatic profile of the region and the practical requirements of spatial thermal comfort assessment in urban planning.

The SSI CALCULATOR plugin facilitates thermal comfort analyses based on temperature and humidity data through an easy, fast, and user-friendly approach. Additionally, it offers users the flexibility to select the unit of input data as either Celsius (°C) or Fahrenheit (°F) and allows the analysis results to be displayed in the preferred unit. This feature differentiates it from existing analyses in QGIS by enabling users to input and output data not only in Fahrenheit but also in Celsius. This innovation enhances the flexibility and usability of the plugin for researchers working with different unit systems. Developed using the Python programming language, the plugin provides a set of integrated functions within QGIS.

This study contributes to the literature in three key ways. First, by developing the first plugin for calculating the SSI within QGIS, it introduces a technical innovation to the open-source software community. Second, it provides a methodological approach that facilitates the spatial analysis of summer temperatures in hot and humid regions, enabling a more effective evaluation of climate data. Lastly, the plugin serves as a decision-support tool by identifying localized areas of high thermal stress, which can guide urban planners in addressing climate adaptation challenges.

## QSSI plugin development and data management

### Adana as an illustrative example

**Fig. 1:**
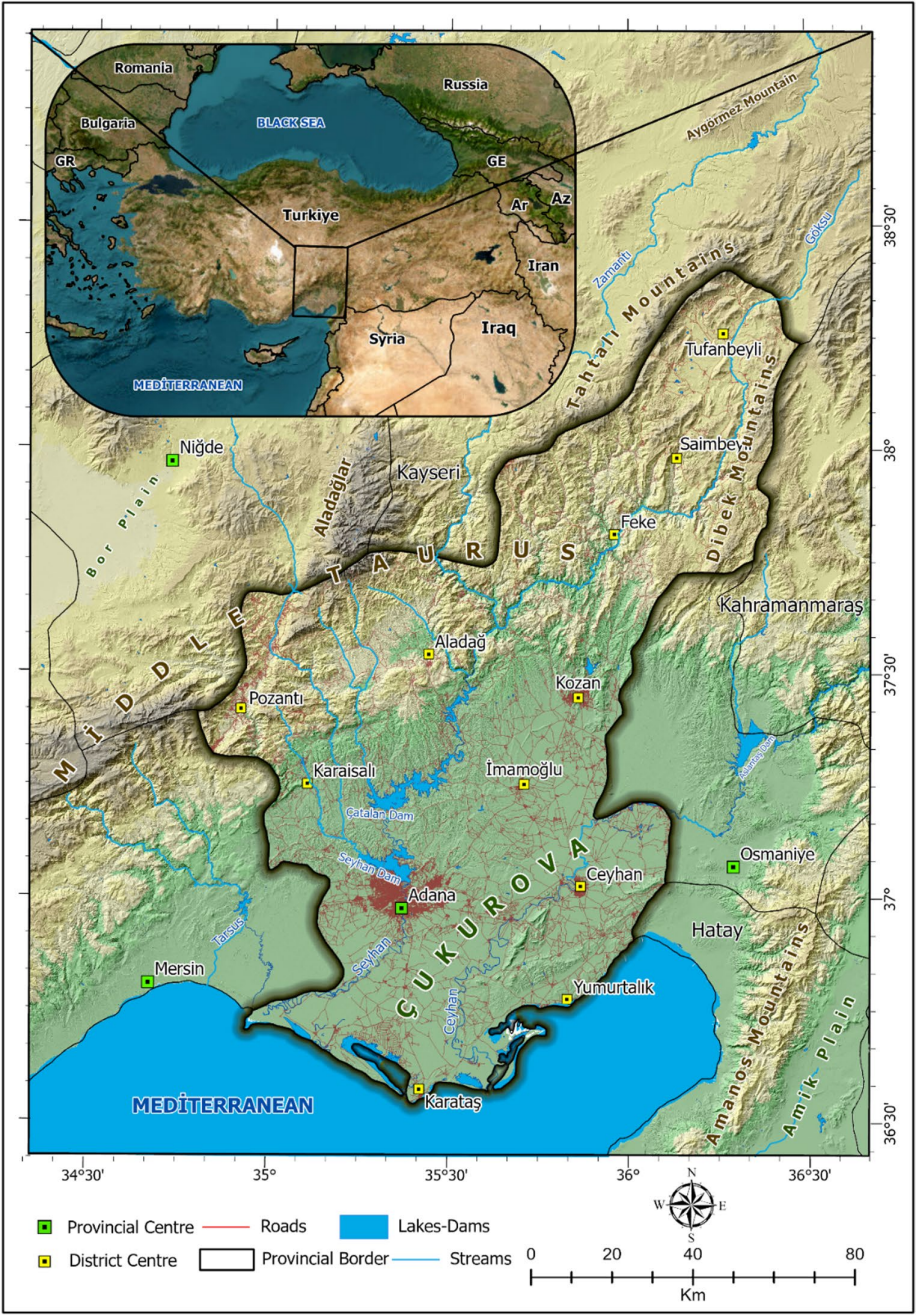
Geographic Location of Adana within Türkiye, Including Major Features

Adana province is selected within the aim of this study. This province is located in the Mediterranean Region of southern Turkey, situated approximately between 37°00′N latitude and 35°19′E longitude. Positioned about 25 m above sea level, Adana is bordered by the rugged Taurus Mountains to the north and east, while its southern part consists of the fertile Çukurova plain (Alphan 2003; Tezel 2020). The province covers an area of 14,030 km², with approximately 49% mountainous terrain, 23% mid-altitude plateaus, and 27% lowland plains (Çevre, Şehircilik ve İklim Değişikliği Bakanlığı n.d.). This physiographic diversity significantly influences temperature and precipitation patterns, creating notable local climate variations across the region (Fig. 1).

Adana exhibits climatic gradients due to the transition from high, steep mountainous areas in the north to flat, fertile lowlands in the south (Tezel 2020). The coastal and inland areas influenced by the Mediterranean Sea experience the typical Mediterranean climate, characterized by mild, rainy winters and hot, dry summers (Göney 1976; MGM 2024). In contrast, the northern mountainous areas, shaped by higher elevations and more continental conditions, are characterized by lower temperatures, higher precipitation, and occasional persistent snow cover (Tezel 2020; MGM, 2024).

In addition to its climatic characteristics, Adana has experienced rapid urban expansion over recent decades, particularly toward the fertile Çukurova plain. This process has led to the conversion of agricultural lands into residential, industrial, and transportation areas, increasing the extent of impervious surfaces while reducing vegetated zones that previously supported local cooling mechanisms (Alphan 2003; Ünal and Uslu 2016; Çilek and Uslu 2022). These land use changes have intensified heat accumulation, limited natural ventilation, and enhanced moisture retention in densely built up areas, thereby amplifying thermal stress during the summer period. Consequently, the observed patterns of thermal discomfort in Adana reflect not only regional climatic conditions but also the long term impacts of urbanization and land use transformation.

Overall, Adana’s climate falls within the Mediterranean type (Csa) according to the Köppen climate classification, indicating hot, dry summers and mild winters (Kum and Çelik 2014; MGM 2024). The annual average temperatures range between 18.7 °C and 19.1 °C, with January being the coldest month (~ 9 °C) and August being the hottest (~ 28–29 °C). The annual precipitation varies between 625 and 671 mm (Tezel 2020; Meteoblue 2024). Relative humidity levels in Adana are generally high, particularly due to humid air mass inflows and the presence of irrigated agricultural areas, where humidity levels can exceed 85% during summer months (Göney 1976; Ünal and Uslu 2016; Altunkasa and Uslu 2020).

In 2023, Adana, with a population of approximately 2.27 million, ranked as the sixth most populous city in Turkiye and served as a significant agricultural and industrial center (Akın et al. 2014; TUIK, 2024). In the context of these longterm land use changes, the spatial patterns identified in this study reveal how urban expansion has translated into distinct thermal stress zones across the city. Areas characterized by dense built-up structures exhibit higher SSI values, indicating that land use transformation plays a key role in shaping the observed distribution of summer thermal discomfort. The high-density urban areas, combined with the proximity of the Seyhan River, irrigation networks, and the Seyhan Dam Lake reservoir, intensify local atmospheric conditions by increasing humidity and restricting wind circulation (Altunkasa and Uslu 2020; Ünal and Uslu 2018).

Given that hot and humid summers are the norm in the region, applying the Summer Simmer Index (SSI) is essential for evaluating Thermal comfort and thermal stress. The combination of specific topography, urban morphology, and local climatic influences intensifies the perception of heat stress; thus, Adana serves as an ideal case study for SSI. Another crucial factor influencing SSI readings is the prolonged summer season, characterized by high relative humidity and minimal wind flow in urban areas (Fig. 1). Consequently, with extensive anthropogenic modifications to land cover across the study area, this major Mediterranean city serves as a prime example for informing urban planning, public health initiatives, and sustainability policies for other urbanized centers in the region.

QGIS is a fully-fledged open source GIS software with strong support from both users and developers. QGIS development benefits from continuous updates by its developers and the addition of new plugins to enhance its functionality. Due to its open-source nature and plugin-based architecture, QGIS offers a suitable framework for implementing the proposed spatial suitability tool. Another notable addition is the QGIS open source style that allows researchers and developers to create plugins tailored to their needs. The software provides a framework under the GNU license service, so researchers and developers can create unique plugins to meet their needs. In addition, hundreds of plugins available in its repositories are freely available to both independent and academic researchers. This work has developed a tool that can calculate the Summer Simmer Index (SSI) quickly and efficiently. QSSI CALCULATOR, after reviewing the basic plugin requirements of QGIS and deciding to create a tool that works seamlessly with the program, we created the “QSSI CALCULATOR plugin”. However, as a data-driven tool for assessing the effects of hot summer weather, QSSI Calculator is not the only solution to this problem. Alternatively, the raster calculator included in QGIS can be used. However, the Raster Calculator requires users to manually construct complex expressions that often involve multiple layers, conditional weights, and normalization factors. As a result, this process becomes prone to user errors, including incorrect syntax, logical miscalculations, and inconsistent parameter inputs. In addition, performing these operations repeatedly for batch analyses or large spatial datasets leads to significant slowdowns in processing time and hampers workflow efficiency. These limitations highlighted the need for a dedicated tool that simplifies the procedure, reduces error potential, and accelerates computation. For this reason, the plugin was developed and integrated into QGIS, a widely used open source GIS software. The graphical user interface (GUI) was developed in a widely used toolkit for GUI programming called Qt Designer and the Python programming language. QSSI CALCULATOR is now a plugin in QGIS, a widely used open source GIS program, and is ready to be used. Once downloaded from the plugin repository, QSSI Calculator does not require any additional operations and is ready to work with QGIS software. After downloading the plugin as it is available in the repository, the necessary code for QSSI Calculator will be available on the computer in question. This allows for full integration of the programming language, which means that modeling related to GIS data formats can be a core part of the main GIS application (Rossetto et al. 2022). With its user-friendly interface, the plugin can easily calculate the Summer Simmer Index (SSI) in a few steps. This is also provided with the plugin, which was developed in python and tested on Windows 11 using QGIS 3.34.

### Data entry

In order to determine the Summer Simmer Index (SSI) characteristics of Adana province, climate data comprising long term temperature (°C) and relative humidity (%) records were obtained directly from the station network of the Turkish State Meteorological Service (MGM). These point based observational datasets were transferred into QGIS to construct the primary spatial database. To transform these discrete station measurements into continuous surfaces representing the entire study area, the Cokriging interpolation technique was selected.

Cokriging was chosen over deterministic methods (e.g., IDW) or Ordinary Kriging because it incorporates a secondary variable (covariate) that is spatially cross correlated with the primary variable to support the estimation process. In this study, elevation derived from a 30-meter resolution Digital Elevation Model (DEM) was employed as the secondary covariate. This methodological choice is critical for Adana’s climate region, which is characterized by sharp topographic transitions from the coastal plains to the Taurus Mountains. Since temperature exhibits a strong physical correlation with altitude (lapse rate), incorporating elevation data allows the model to predict thermal patterns in areas with sparse station coverage more accurately than methods relying solely on distance.

While Cokriging is computationally more demanding and requires a strong statistical correlation between the primary and secondary variables to be effective, its ability to reduce prediction error variance in topographically complex regions outweighs these limitations (Cetin et al. 2018; Konomi et al. 2023). By leveraging the high resolution DEM, the final temperature and humidity raster maps were generated at a 30-meter spatial resolution. This resolution ensures that the resulting SSI maps capture local microclimatic variations driven by topography, providing the necessary detail for district-level thermal comfort assessment and reproducibility of the workflow.

**Fig. 2:**
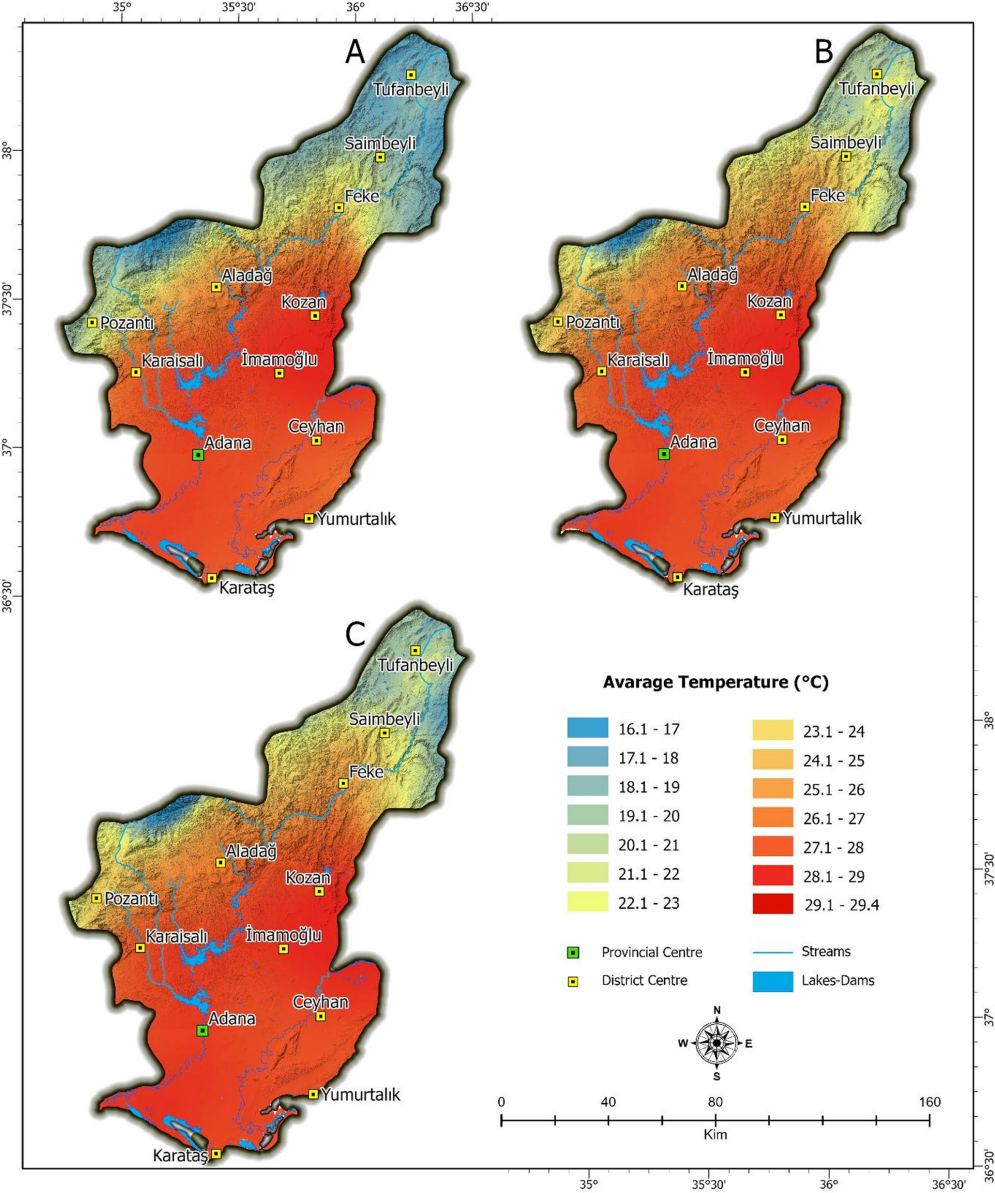
Spatial Distribution of Temperature in Adana: (**A**) June, (**B**) July, (**C**) August

**Fig. 3:**
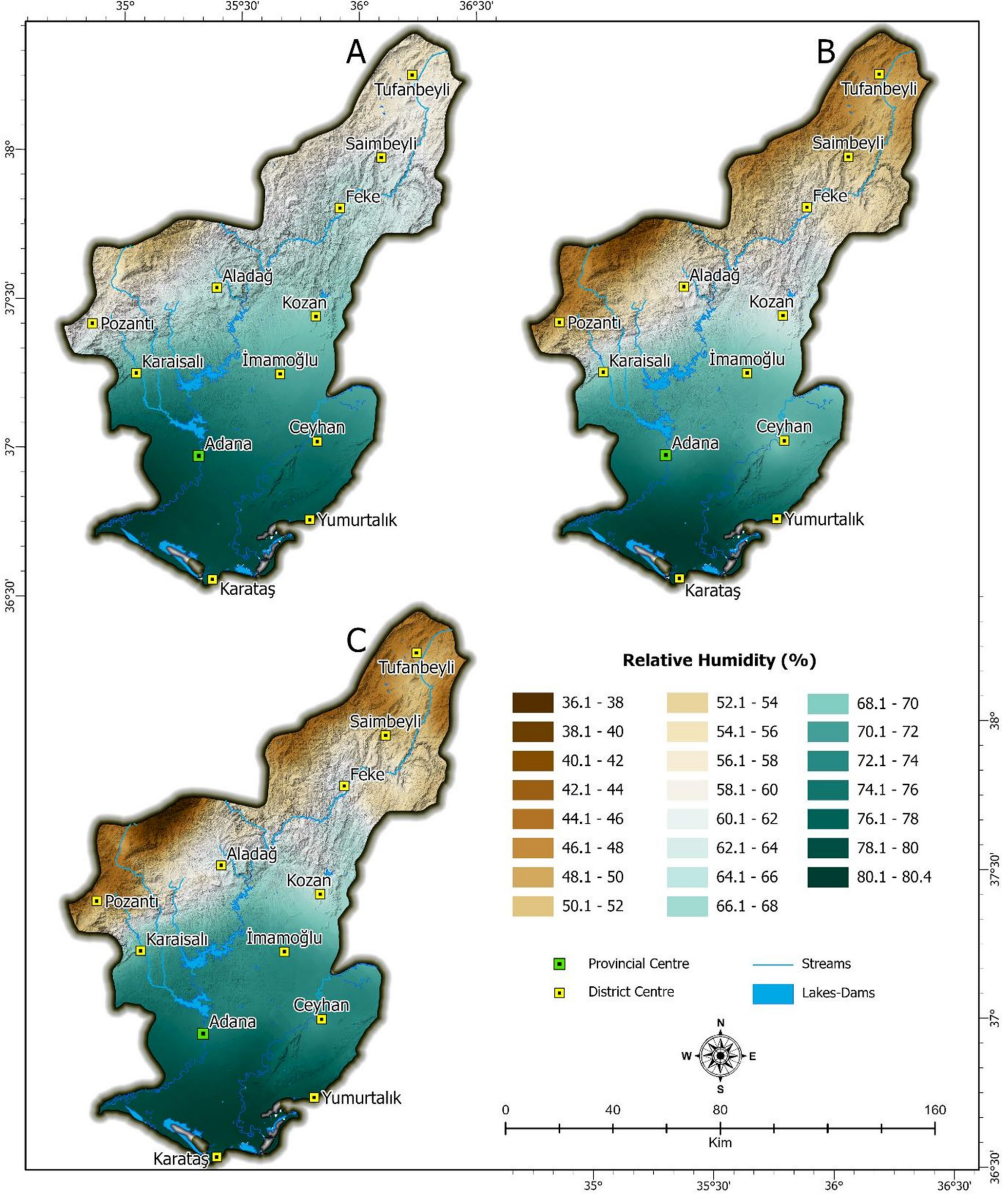
Spatial Distribution of Relative humidity in Adana: (**A**) June, (**B**) July, (**C**) August

Adana exhibits the characteristic climatic features of the Mediterranean region. In this context, the summer months in the study area are hot and dry. Temperature and relative humidity patterns were analyzed at a regional scale to capture broad spatial gradients across Adana province, particularly the contrast between northern highlands and southern lowland urban areas. While these visualizations provide insights into meso-scale climate behavior, their implications for thermal comfort within the urban fabric necessitate finer resolution analysis. The current version of the QSSI tool operates at a neighborhood-to-district scale based on available raster data resolution (e.g., 30 m), which allows general identification of thermal stress zones. However, the tool is designed to be extendable, and further integration with higher-resolution urban datasets (e.g., building-level or sensor-based microclimate data) is anticipated for future versions. A more detailed discussion of scale transitions-from regional observation to human-scale decision-making-is included in the discussion section to address this issue. (Figures 2 and 3). This difference creates a distinct north-south transition, varying the temperature between 16.1 °C and 29.4 °C. This distribution shows that the warm ambient conditions of the Mediterranean climate are more pronounced especially in the lowland and coastal regions where the city of Adana is located (Figs. 2 and 3). Parallel to the temperature distribution, there is a large difference in the relative humidity distribution between 36.1% and 80.4%. In the high altitude regions in the north, low temperatures and reduced evaporation rates often result in humidity ranging from 36% to 50%. In contrast, in the southern Cukurova and coastal regions, where the city of Adana is located, the relative humidity ranges from 66% to 80%, showing a significant increase. In fact, the relative humidity exceeds 80% along the Mediterranean coastal zone, including the Karataş district, where the maritime influence is strongly felt. The combination of high temperatures and high humidity in the lowland and coastal areas exacerbates urban heat island effects, leading to a concentration of severe thermal discomfort in the city center. While the cooler and drier northern regions of the province offer more favorable climatic conditions, the topography and rich biodiversity (Karadeniz 2024) are not suitable for the sprawl of a large city like Adana. However, due to the decrease in thermal comfort in the plain during the summer, many people move towards the more comfortable and mild climatic conditions in the north. In particular, these daily movements occur due to increased heat stress in densely populated urbanized areas (Sevgi 1984; Karagel ve Karagel 2010).

### Model implementation and operating architecture

**Fig. 4:**
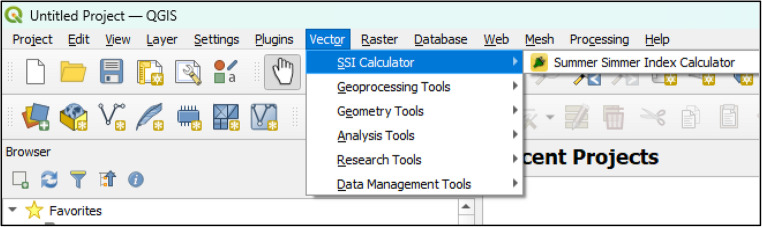
The graphical user interface of QSSI plugin

Since the data are prepared in the required file formats, the first step involves loading the.tif format images into QGIS. To ensure efficient use of the plugin, access is provided by integrating it into the QGIS graphical interface through the QGIS Processing Toolbox. QGIS software natively supports the Python programming language for plugin development. Accordingly, the QSSI plugin was implemented using Python within the architectural and technical framework defined by QGIS. The graphical user interface (GUI) of the plugin was designed using Qt Designer, a Python-compatible interface development framework that enables the creation of tool-based interfaces for QGIS plugins. Qt Designer facilitates seamless integration between the visual interface and Python-based backend processes Fig. 4.

**Fig. 5:**
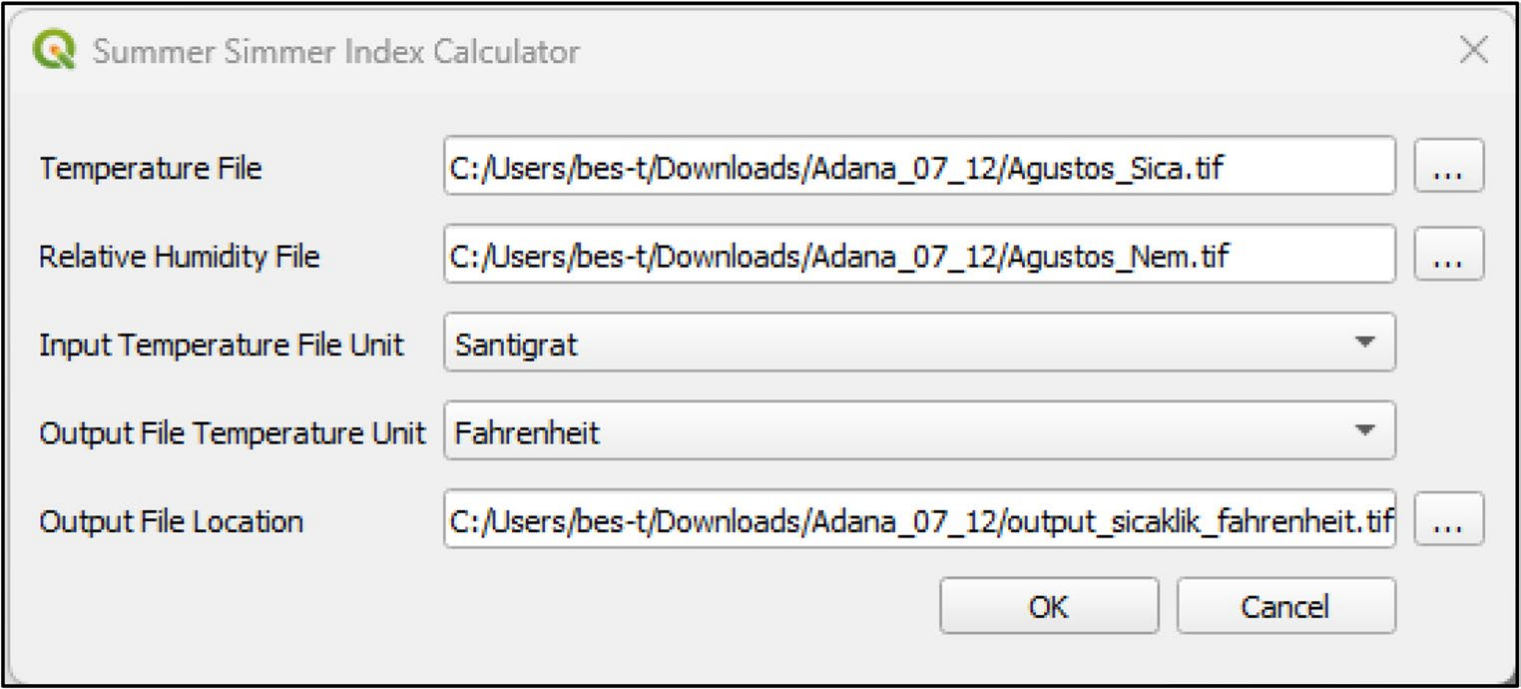
Data Entry Interface of QSSI Plugin

The main graphical interface of the plugin consists of two input fields, two selection fields, and one output selection field. The first input field is used for the temperature raster data, while the second input field is designated for the humidity raster data. The “Input Temperature File Unit” selection field specifies the default temperature unit of the provided temperature data. The “Output Temperature Unit” selection field determines the temperature unit for the resulting map layer after the calculation. The “Output File Location” field designates the directory where the output map will be saved. Through this interface, users can input temperature and humidity maps, select the temperature unit used in the input datasets, and define the temperature unit of the output map. These selections enable flexible handling of different data sources and ensure consistency between input and output datasets. For example, when performing a calculation using a temperature raster data in Fahrenheit, the “Input Temperature File Unit” must be set to Fahrenheit. If the final output values and map should be in Celsius, the “Output Temperature Unit” must be set to Celsius. Once the necessary selections are made, clicking the “Confirm” button triggers the predefined computational processes coded into the plugin already Fig. 5.

**Fig. 6:**
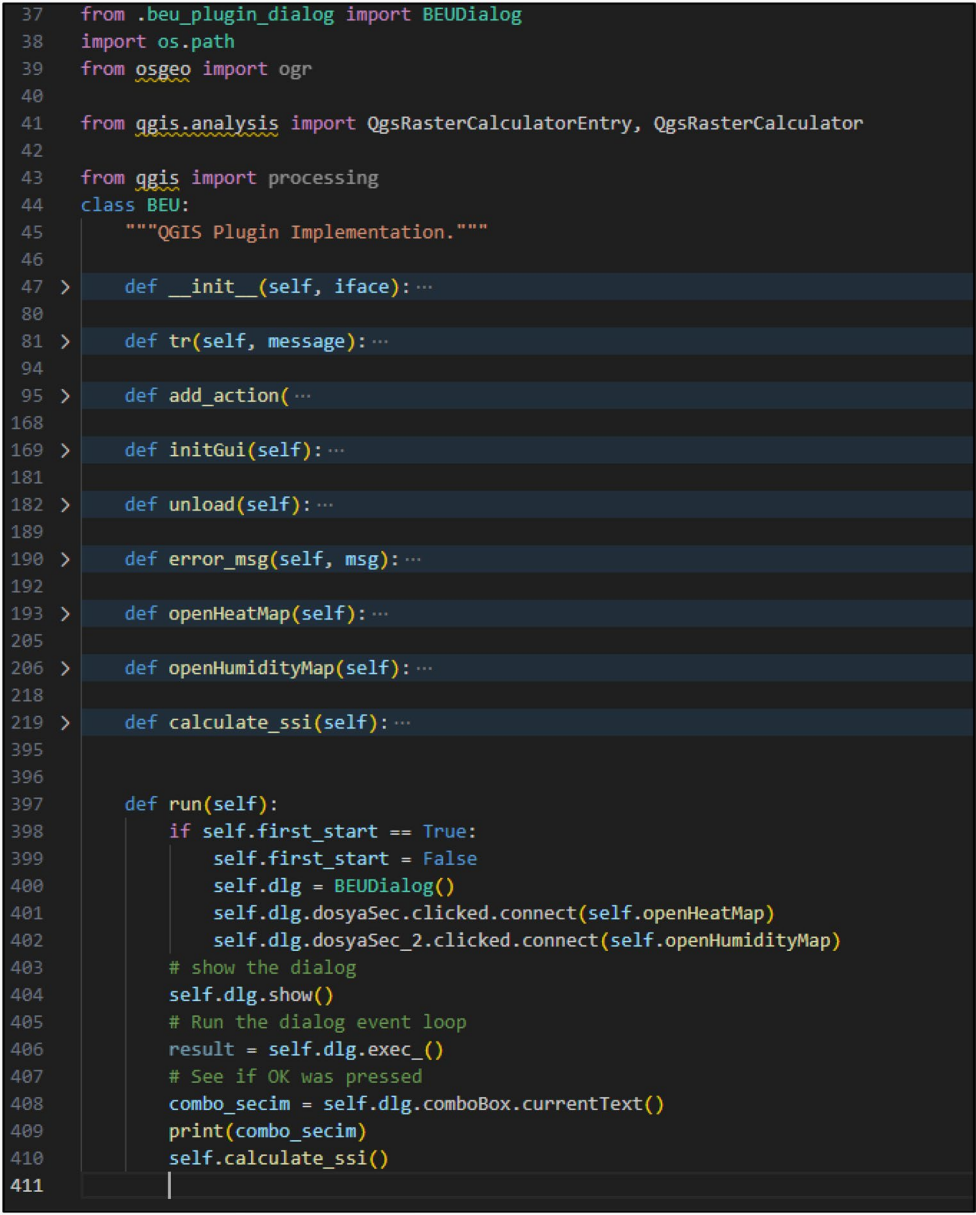
QSSI Plugin Python Analysis Code Interface

All interactions initiated through the graphical interface are managed by Python-based backend code. Python is responsible for executing the user-triggered processes, performing the required calculations, and generating outputs in formats compatible with QGIS. In the developed plugin, the processed output is produced as a.tif file and saved to the user-defined file location specified in the interface. The script triggered by the “Confirm” button is structured as follows. It consists of one class and five functions. The openHeatMap and openHumidityMap functions enable the selected temperature and humidity maps to be displayed within the layers widget in QGIS. The calculate_ssi function performs the index calculation as described in the study, using the temperature and humidity maps as input data. The script then adds the calculated layer to the layers section and saves the output map in.tif format to the specified file path. At this stage, the user must select the output layer. This integrated design allows the QSSI plugin to effectively combine user interaction, data processing, and computational analysis, resulting in a functional and sustainable analytical tool operating entirely within the QGIS environment Fig. 6.

### Spatial relationship between SSI zones and demographic structure

To assess how thermal stress affects different population groups, the 2024 neighbourhood population data of Adana were integrated into the SSI analysis. Population figures obtained from the Turkish Statistical Institute (TÜİK, Address Based Population Registration System) were joined with neighbourhood boundary polygons provided by the Adana Metropolitan Municipality. After harmonizing the coordinate systems, an Overlay Analysis was performed to determine the number of residents falling within each SSI category. This process enabled a direct comparison between population density and thermal stress intensity across the province. The resulting exposure patterns are presented in Table 2; Figs. 8 and 9.

## Application of QSSI and results

### Case study application and model validation

**Fig. 7:**
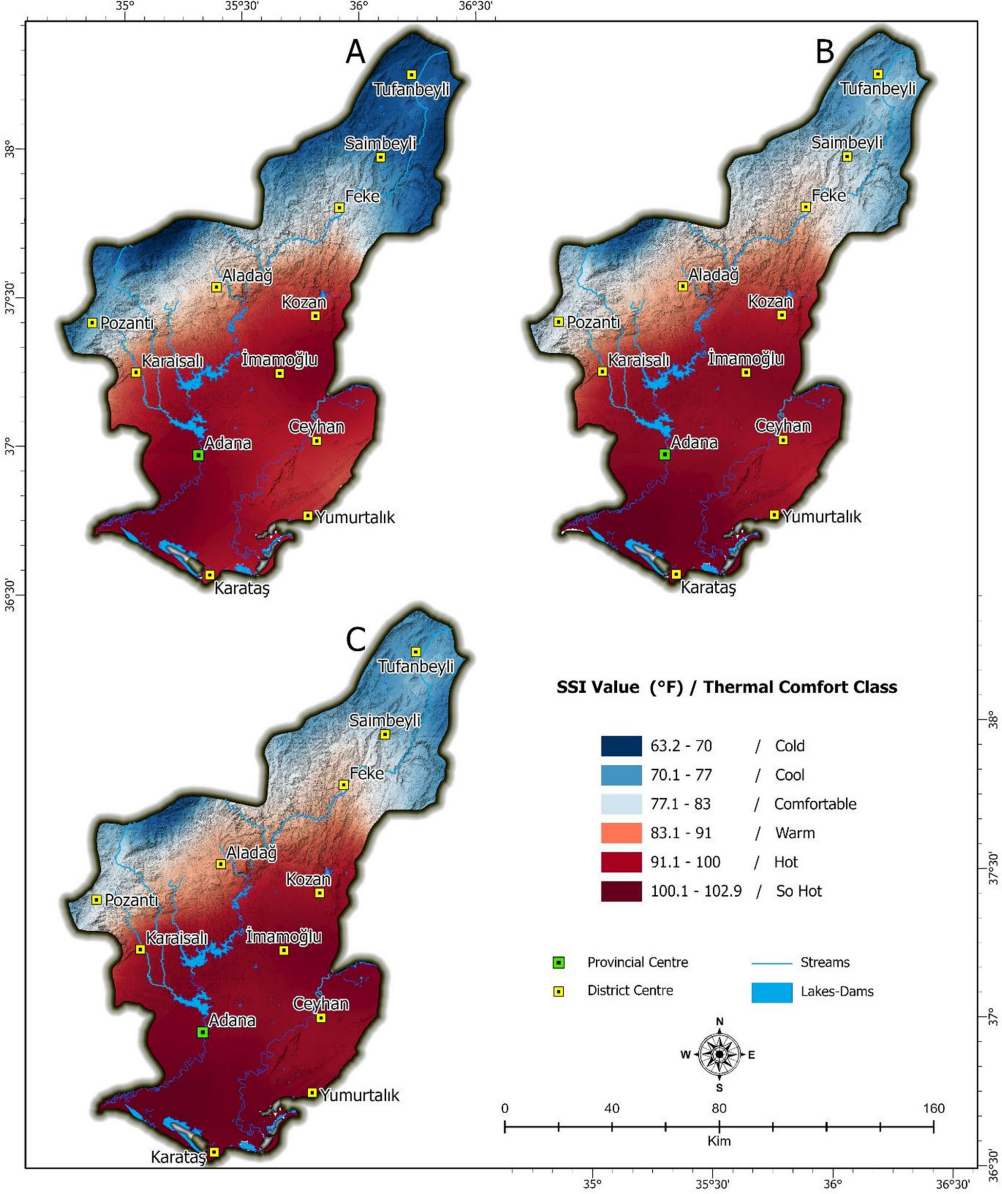
Thermal Comfort Conditions in Adana: (**A**) June, (**B**) July, (**C**) August

The results of SSI analyses conducted across Adana for June, July, and August, based on temperature and relative humidity data, are presented in Figure (7). According to the climatic classification results, in June, 38% of the study area falls within the “Cold” category, 50% in the “Comfortable” category, and 12% in the “Warm-Hot” category. In July, this distribution changes to 21% “Cold,” 17% “Comfortable,” 41% “Warm-Hot,” and 21% “Sweltering.” By August, the proportions shift further, with 22% “Hot,” 15% “Comfortable,” 26% “Warm-Hot,” and 37% “Sweltering” areas.

Based on the meteorological data from 1985 to 2024, the results indicate that large urban areas are highly unfavorable in terms of thermal comfort. The perceived temperature analyses for the study area were conducted using the SSI formula, and SSI maps were generated accordingly. The SSI maps were created using the following formula: (Pepi 1987a, b)

$$SSI=1.98\left[T_a-\left(0.55-0.0055U_r\right)\left(T_a-58\right)\right]-56.83$$

In this formula, temperature values are calculated in degrees Fahrenheit (°F). To apply the formula, temperature maps in degrees Celsius (°C) were converted to °F using QSSI (Adıgüzel et al. 2022). The analysis results show that SSI values exceeding 100 °F indicate temperatures that pose significant health risks. As seen in Table 1, areas with SSI values above 100 °F are potentially hazardous for human health, and prolonged exposure in these regions may lead to severe or even fatal consequences.

**Table 1:** Interpretation of SSI values(Asghari et al. 2021)

SSI value (^0^F)	Sensed Thermal Comfort	Type
70 ≤ SSI < 77	There is a cosy atmosphere for most people, but it is a bit cool.	Cold
77 ≤ SSI < 83	It is the ideal temperature for almost everyone and everyone feels comfortable	Cold
83 ≤ SSI < 91	A favourable environment for most people, but feels a little warm	Comfortable
91 ≤ SSI < 100	Temperatures above normal values	Warm-Hot
100 ≤ SSI < 112	Various disorders occur due to prolonged exposure	Sweltering
112 ≤ SSI < 125	For almost everyone, the perceived temperatures have high values. Various diseases occur with prolonged exposure	Extremely Hot
125 ≤ SSI < 150	It has temperatures that affect the vital functions of particularly vulnerable groups (patients, the elderly and children).	Extremely Hot
150 < SSI	Fatal consequences in case of prolonged exposure	Deadly Hot

While Table 1 presents the standard interpretation of SSI values based on established literature, similar thermal stress categories have been associated with documented health impacts in Mediterranean climate cities. Previous studies have demonstrated that SSI classifications provide a meaningful representation of perceived thermal stress under hot and humid conditions, including Mediterranean environments (Asghari et al. 2021; Ghalhari et al. 2022). Moreover, empirical evidence from Mediterranean cities indicates that increasing thermal stress driven by urban warming is directly linked to adverse health outcomes, including elevated heat related mortality risks during periods of prolonged exposure to high temperature and humidity (Pyrgou and Santamouris 2018). In this context, the SSI thermal stress categories applied in the present study allow the results obtained for Adana to be interpreted within a broader Mediterranean and public health framework.

After calculating the SSI values for the study area using QGIS, the thermal comfort zones for the summer months (June, July, and August) in the Northern Hemisphere are presented in Fig. 7. The analysis indicates that the northern part of the study area falls within the 70 ≤ SSI < 90 °F range during June, July, and August. These regions experience the most favorable climatic conditions for nearly all individuals. In contrast, the southern part of the study area remains outside the thermal comfort zone throughout the summer months, exhibiting highly unfavorable conditions. The SSI values in these areas range from 91.1 ≤ SSI < 102.9 °F, indicating that perceived temperatures are significantly above normal levels, which poses adverse effects on human health. These results show the quantitative distribution of thermal comfort zones in Adana province during the summer months. These comfort zones are not caused by climatic differences during the summer months. Looking at the geographical features of the area where Adana was founded, it can be seen that the comfort zones change from south to north. In particular, topography, urban land use density and general land use type cause these comfort zones to vary. Although the dams and lakes in Adana create more suitable thermal comfort zones in areas with cultivated and irrigated agricultural land, it has been determined that thermal comfort decreases in densely urbanised areas. In particular, heat accumulation is increasing in the urban area constructed due to intensive development, insufficient green space and uncontrolled urban development, especially in the city centre of Adana. This situation causes an increase in the temperature felt, particularly during the summer months. Furthermore, the energy released as a result of anthropogenic activities also contributes to a decrease in thermal comfort. In contrast, it is observed that the rural settlements located to the north of the study area have lower levels of urban development and, due to topographical reasons, enjoy more favourable thermal conditions.

**Fig. 8:**
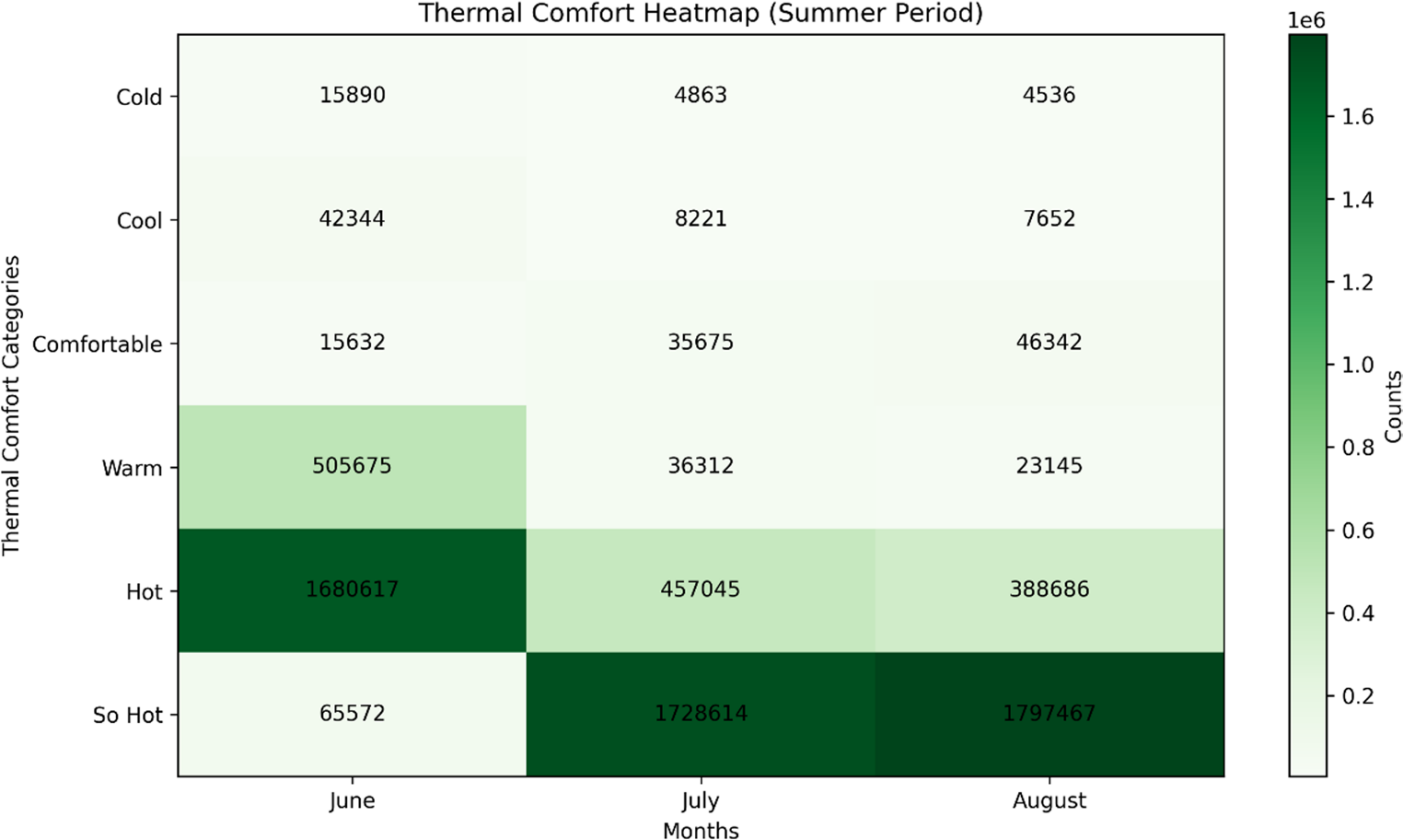
Thermal Comfort Heat Map for Adana (Summer Period)

The results concerning the spatial distribution of thermal comfort are closely related to Adana’s locational characteristics and urbanisation process. However, individuals living in rural and urban areas within the province’s borders are clustered in a highly disproportionate manner. In this context Fig. 8 illustrates the relationship between the spatial distribution of the population and thermal comfort zones. The results indicate that no settlement area falls within the range of 112 ≤ SSI < 150 °F, which represents temperatures that could cause fatal health conditions. However, approximately 80% of the total population resides in “Hot” and “So Hot” zones. The primary reason for this distribution is the spatial concentration of the population within Adana. Although the total area of Adana province covers 13,844 km², nearly 80% of the population resides within the 1,945 km² urban core of Adana city center (Table 2). The SSI values within Adana’s urban areas range between 100.1 °F and 102.9 °F. Given that 1,800,619 people live in the densely populated urban area, the high population density and built environment significantly amplify the difference between perceived temperature (SSI) and actual air temperature, leading to intensified thermal stress during summer months.

**Table 2:** Distribution of Thermal Comfort Categories During Summer Periods

Thermal Comfort Category	June (%)	July (%)	August (%)
Cold	0.7	0.21	0.2
Cool	1.86	0.36	0.34
Comfortable	0.69	1.57	2.04
Warm	22.27	1.6	1.02
Hot	73.99	20.13	17.12
So Hot	2.89	76.14	79.28

**Fig. 9:**
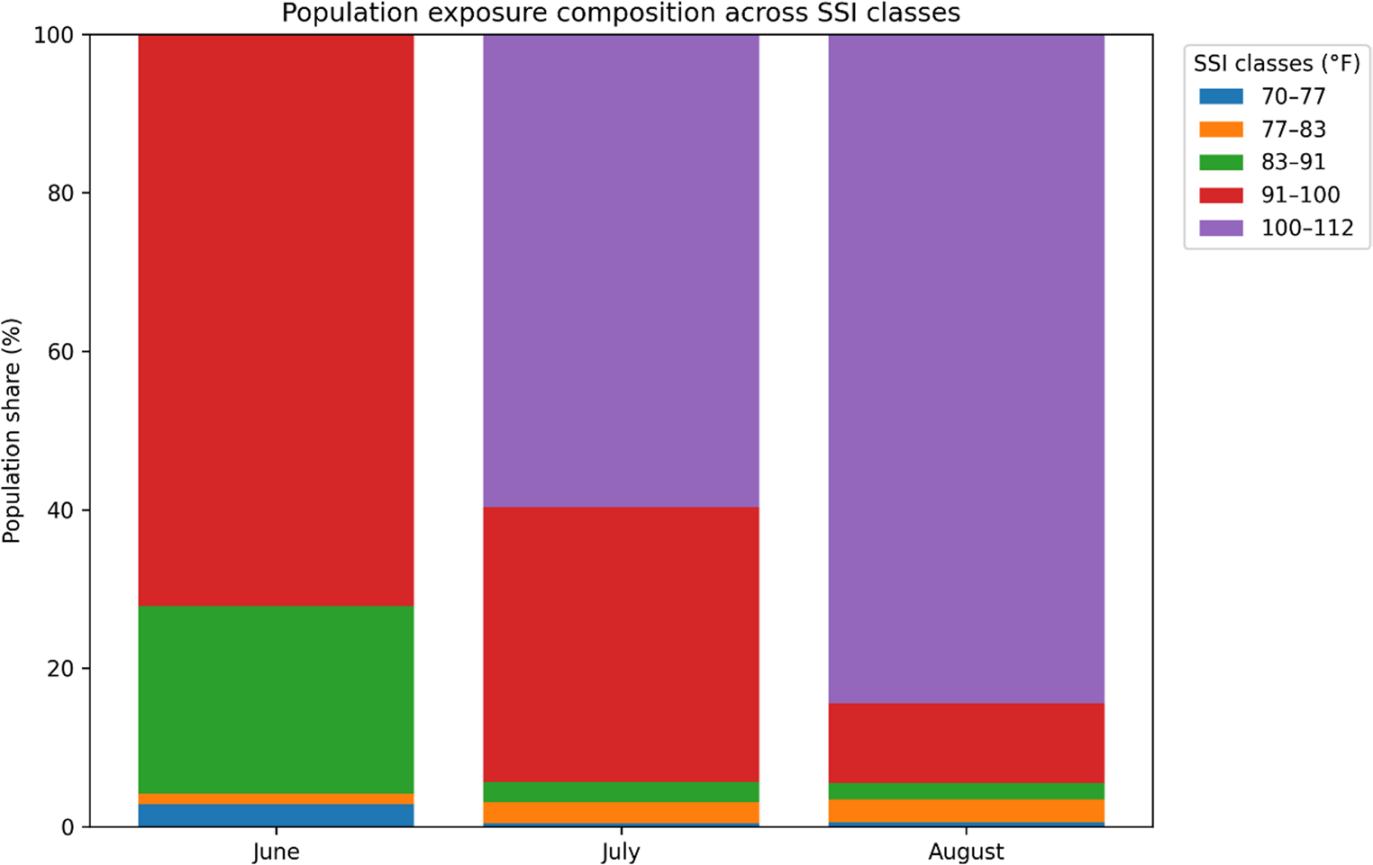
Population exposure to SSI classes in Adana

An examination of Adana’s thermal comfort zones during the summer season reveals that less than 1% of the total population resides in “Cold” areas across all three months. Interestingly, the “Comfortable” category, which represents the most favorable thermal comfort conditions, shows a gradual increase in proportion from June to August, yet only around 2% of the total population experiences these conditions. In June, approximately 22% of the population falls within the “Warm” category. However, as temperatures peak in July and August, this proportion decreases by 1%. Meanwhile, 74% of the population experiences the “Hot” category in June. As temperatures rise in July and August, individuals residing in these zones experience a significant decline in thermal comfort. By the peak of summer, over 79% of the total population is exposed to the “So Hot” category, which poses a high risk of heat-related illnesses with prolonged exposure (Fig. 9).

These findings indicate that Adana predominantly falls within the “Hot” and “So Hot” thermal comfort categories during the summer months. Changing climate conditions pose a significant risk to the majority of the population, and without preventive measures, more severe challenges may arise in the coming years. In areas with low thermal comfort, vulnerable groups such as the elderly, children, and individuals with chronic illnesses face a heightened risk of life-threatening health impacts. Beyond its direct consequences for human health, this issue also intensifies challenges related to energy demand, agricultural productivity, and water resources, ultimately undermining climate resilience. In highly urbanized areas with a Mediterranean climate, such as Adana, efforts to combat changing climate conditions must be prioritized. To enhance urban resilience-a city’s capacity to absorb, recover from, and adapt to climate-related stressors -it is essential to implement targeted adaptation strategies, such as increasing vegetative cover, improving ventilation corridors, or optimizing urban morphology to reduce heat stress. These actions address exposure and vulnerability, key dimensions in climate resilience frameworks. Unlike mitigation strategies that aim to reduce greenhouse gas emissions, these localized interventions aim to adapt urban environments to the immediate impacts of extreme weather events, such as prolonged summer heat.

## Discussion

This study set out to address two primary objectives: to develop an open-source GIS tool for automating Summer Simmer Index (SSI) calculations and to analyze the spatial distribution of thermal stress in a dense Mediterranean city. The findings directly respond to these research goals. Firstly, the successful development and implementation of the QSSI Calculator demonstrate that bioclimatic analysis can be effectively integrated into the QGIS environment, filling a technical gap in open source spatial planning tools. Secondly, the case study in Adana reveals that rapid urbanization has created distinct thermal stress zones, with the city center experiencing significantly higher SSI values compared to rural surroundings. These results are particularly significant when considered in the context of global climate trends.

According to recent studies, global climate change is predicted to trigger considerable changes in climatic parameters in the near future with or without magnification effect on all living organisms on Earth (Cetin et al. 2023; Koç 2022). The increase in temperature and humidity (Cetin et al. 2018) is expected to cause significant changes in Thermal comfort zones (Cetin et al. 2018). Hence, the ability to adjust the thermal comfort zones for varying climate or even designing a fitting urban and environmental method is critical (Jia et al. 2022). In this context, the SSI CALCULATOR plugin provides a rigorous spatial analysis of thermal stress during summer, offering insights into the spatial distribution of heat, which can serve as a foundation for these designs. The mapping of bioclimatic stress zones based on heat and humidity may be among the key instruments to shape the most effective intervention strategies to fight climate change in the long run.

Adana, characterized by high temperatures and humidity levels typical of the Mediterranean climate, is a city where significant Thermal comfort losses occur. The analyses conducted in this study, based on temperature and relative humidity data, reveal the spatial distribution of thermal stress zones during the summer months. The findings indicate that the thermal stress is significantly high in densely urbanized areas, placing them predominantly within the “Hot” and “So Hot” categories. Notably, approximately 80% of the population residing in Adana’s urban core experiences these extreme conditions during the summer months. This observation aligns with existing studies highlighting the strong relationship between urbanization and the urban heat island effect (Cetin et al. 2024; Kawamoto 2017; Ren et al. 2022; Santamouris et al. 2015). Furthermore, the results reinforce previous research on the negative health impacts of rising temperatures (Ren et al. 2022; Schlegel et al. 2020; Vanos et al. 2010).

Additionally, a comparative analysis between Adana’s urban and rural areas based on SSI values demonstrates the significant influence of urbanization on Thermal comfort. While lower SSI values were observed in rural regions, the protective effect of vegetation cover was evident. In contrast, higher SSI values in urbanized areas clearly illustrate the adverse effects of urbanization on Thermal comfort. These findings not only provide empirical evidence for Adana but also serve as a valuable guide for developing broader policies in urban planning and public health.

The examination of existing GIS and QGIS-based thermal comfort and bioclimatic analysis tools clarifies the position of the SSI CALCULATOR plugin in the literature. Tools commonly used in the QGIS ecosystem mostly address outdoor thermal comfort using microclimate and radiation-based modeling approaches. For example, UMEP is an urban environment analysis system that includes a series of models and tools within QGIS. Among these, the SOLWEIG model calculates short- and long-wave radiation to produce the mean radiant temperature (Tmrt) (Lindberg et al. 2018; Mutani and Beltramino 2022). This Tmrt output from SOLWEIG, combined with UMEP’s thermal comfort modules, enables the calculation of PET and similar thermal comfort indices (Mutani and Beltramino 2022). Similarly, UMEP-based tools such as TreePlanter are models for optimizing tree layout to reduce thermal heat stress based on the represented radiation load (UMEP Development Team. n.d.; Wallenberg et al. 2022). Other QGIS plugins generate near-surface air temperature maps but do not provide direct measurements of perceived thermal stress specific to the summer season (Touati et al. 2020). Furthermore, many workflows rely on external modeling environments such as RayMan for PET and UTCI analyses (Jänicke et al. 2021). In this context, the SSI CALCULATOR plugin offers a complementary and operational approach. The plugin calculates SSI, a heat-focused thermal stress indicator, directly within the QGIS environment using only temperature and relative humidity data as inputs. This approach significantly reduces data requirements and analysis complexity compared to multi-parameter and complex modeling processes, enabling rapid and practical spatial assessments, particularly in hot and humid regions.

The QSSI Calculator plugin represents a significant contribution to the literature, as it is the first QGIS plugin capable of conducting spatial Summer Simmer Index (SSI) calculations. Existing studies (Adıgüzel and Doğan 2021; Arıcak 2020; Asghari et al. 2021; Pepi 1987a, b; Sancar and Güngör 2020) indicate that SSI analyses have traditionally been performed using manual calculations with limited spatial coverage. By overcoming these limitations, the QSSI Calculator plugin enables rapid, large-scale, and user-friendly SSI analyses. This innovation is particularly valuable in hot and humid regions, as it allows for a detailed assessment of thermal stress and provides a new methodological framework for sustainable urban planning and public health research. Furthermore, the plugin’s ability to support both Celsius (°C) and Fahrenheit (°F) inputs enhances its usability for researchers working accross different geographical regions.

Compared to other bioclimatic indices such as UTCI and PET, the SSI CALCULATOR plugin distinguishes itself by its specific focus on summer temperatures. While UTCI and PET are widely used across various climate conditions, SSI’s ability to provide detailed summer-specific analyses allows for more precise assessments in hot and humid regions. For instance, although PET, developed by Matzarakis et al. (1999) and Höppe (1999a), is a comprehensive index for evaluating overall thermal comfort, it does not emphasize specific summer stress factors as effectively as SSI. In this regard, the QSSI Calculator plugin fills this gap in the literature by integrating SSI calculations into QGIS, making them accessible in a spatial context. This capability positions the plugin as a comprehensive tool not only for measuring thermal stress but also for informing long-term urban adaptation strategies.

## Theoretical and practical implications

### Theoretical implications

QSSI CALCULATOR plugin adds new value to existing theoretical background relevant for Thermal comfort analyses. The integration of bioclimatic indices with spatial analysis remains scarce in the existing literature. This study introduces a novel methodological and theoretical approach to bioclimatic analysis, demonstrating that the calculation of the Summer Simmer Index (SSI) can be effectively applied in a spatial context In addition, the use of SSI combined at local level from calculations with spatial visualization provides a different view of thermal comfort. This model can be a norm for all future researchers and urban planners to better bioclimatic analyses at more local scales.

### Practical implications

In practice, the QSSI Calculator plugin serves as a highly valuable tool for urban planning, public health, and climate change adaptation strategies. The AC simulation studies were successfully applied to the specificity of Adana and the plugin have provided a spatial distribution about thermal stress clearly, helping the addressing strategies for increasing bioclimatic levels of comfort in susceptible areas. The spatio-temporal analyses produced by the QSSI plugin provide insights at a neighborhood and district scale, which can support local interventions to reduce the impacts of urban heat islands. While not designed for macro-level urban policy-making, the plugin helps inform localized adaptation strategies-such as identifying heat-vulnerable zones or prioritizing greening efforts within the operational scope of municipal decision-making. By aligning the analytical scale of the tool with the granularity of interventions, it supports data-informed decisions that are practical and context-specific.”

The open-source plugin enables same analysess from various climatic regions across the World, thereby making its use more widespread. The flexibility of QSSI Calculator allows data to be entered in both Celsius and Fahrenheit units, enabling better integration of diverse data sources. This flexibility indicates that the plugin is not only a valuable tool for academic research but also beneficial for urban planning at both practical and local government levels.

## Limitations and future studies

In this study, SSI analyses were conducted solely based on temperature and humidity data. The absence of bioclimatic parameters such as wind speed, solar radiation, and land use presents an opportunity for expanding the scope of Thermal comfort analyses. However, this limitation does not hinder the primary objective of the study, which is to identify bioclimatic stress zones caused by temperature and humidity during the summer months. By design, SSI primarily focuses on temperature and humidity as the most significant stress factors, providing a sufficient foundation for this analysis. Therefore, the study’s findings remain valid within the defined scope of SSI, and this limitation does not compromise the reliability of the results. However, for a more comprehensive bioclimatic assessments, future studies should incorporate wind speed, solar radiation, and other relevant parameters.

The QSSI Calculator Plugin was specially developed to function within QGIS. Although this assures compatibility with open-source GIS platform, in line with the study’s objectives, it also highlights the need for broader adaptation to general GIS softwares. In order to enable the plugin’s use across multiple platforms, extending its capabilities to function in other GIS environments could enhance its accessibility and support cross-platform functionality. Additionally, extending time series support in the plug-in would be beneficial for more accurate long-term climate change projections and extended temporal analyses.

Although this study focuses on a Mediterranean context, the SSI algorithm can potentially be applied to other regions characterized by high temperature and humidity, such as tropical or subtropical climates. Moreover, as climate change is expected to prolong warm periods in many regions, the plugin may also be useful for monitoring heat stress during transitional seasons, including spring and autumn. Nevertheless, it should be noted that the current implementation is specifically designed to assess heat-related stress and does not account for cold stress or winter conditions, which remain beyond the scope of this study.

Finally the changes brought by the improved SSI Calculator plugin already take place well beyond better spatialising of SSI. Acknowledging of limitations in this study represent a basis for future systematic bioclimatic analyses. Future work will prioritize a more targeted and theory driven analysis of key bioclimatic variables, structured around a clearly defined theory of change that links environmental indicators with actionable planning strategies. Rather than broadening the scope, the focus will be on identifying causal pathways that inform sustainability oriented interventions at the urban scale.

## Conclusion

This research focuses on the development and demonstration of the QSSI Calculator, an open source QGIS plug-in for Thermal comfort assessment with the Summer Simmer Index (SSI). This tool, which was developed to enable accurate, reproducible, and user-friendly Thermal comfort analysis and mapping, is intended to support urban planning processes with open-access data and consistent methodologies. During the development phase of the tool, QGIS GIS software was preferred due to its accessibility and flexibility, and an embedded Python-based module was created with a single integration.

This plugin, which maps the Thermal comfort of a region using temperature and relative humidity data in raster format, uses optimized algorithms for high accuracy and computational efficiency. In this way, the plugin provides a fast method for application area comfort analysis with a simple interface. In addition, to optimize the plugin and test its accuracy, the province of Adana was selected, where thermal stress events are severe. This area, which has a Mediterranean climate characterized by high temperature and high humidity during the summer period, was analyzed with the plugin and valuable results were obtained. When these results obtained using the QSSI Calculator are analyzed, it has shown its effectiveness in identifying bioclimatic stress areas associated with temperature and humidity. The study also highlights urban adaptation plans for climate change, highlighting comfort risks for cities where urbanization is concentrated in specific areas, such as Adana. While the plugin can technically be applied to both rural and urban contexts, the primary aim of this study was to assess urban-scale bioclimatic stress and inform adaptation planning in densely populated areas.

The open-source nature of the plugin provides a solid foundation for the development of future climate analysis applications, while its integration into the QGIS architecture supports data-driven policy making for both researchers and decision makers targeting climate adaptation. Future iterations of the QSSI Calculator may offer enhancements in bioclimatic analysis by incorporating additional variables or improving resolution and usability. While further development is needed, its integration within an open-source GIS environment offers potential for broader applicability in climate adaptation planning, especially at the local scale.

## Supplementary Information

Below is the link to the electronic supplementary material.

Supplementary Material 1 (DOCX 15.3 KB)

## Data Availability

Software name: QSSI (QGIS Summer-Simmer Index) Calculator Plugin, Developer: Mansur Beştaş, Fatih Adıgüzel, Enes Karadeniz, Aşır Yüksel Kaya and Rukiye Gizem Öztaş Karlı, Year first available: 2025, Hardware requirements: PC, Software requirements: QGIS 3.x, System requirements: Windows, Linux, Mac, Program language: Python3, Availability:https://github.com/digifelis/summer_simmer_index_gis_plugin
