# Supplementary material for: QSSI (QGIS summer simmer index) calculator plugin: an open-source tool for thermal comfort analysis in gis applications
Source: Int J Biometeorol. 2026 Mar 24;70(4):99. doi: 10.1007/s00484-026-03180-x (PMC13013210; doi:10.1007/s00484-026-03180-x)
Supplement: Supplementary file 1 — Supplementary Material 1 (DOCX 15.3 KB) [file 484_2026_3180_MOESM1_ESM.docx]

**Table 1:** Supplementary Table S1. Comparison of the QSSI Calculator with selected GIS-based thermal comfort studies.

| Research Focus & Tools | Relevant Literature | Limitations / Constraints | QSSI Calculator Innovation (This Study) |
| --- | --- | --- | --- |
| Microclimate & Radiation Modeling (UMEP, SOLWEIG) | Lindberg et al., 2018; Wallenberg et al., 2022 | Requires intricate preprocessing, complex radiation models, and detailed 3D urban data. | Eliminates complex modeling by focusing strictly on air temperature and relative humidity. |
| Multi-Parameter Indices (PET, UTCI, RayMan) | Matzarakis et al., 2007; Jänicke et al., 2021 | Often relies on external modeling environments and involves testing various human factors. | Embedded natively in QGIS; specifically tailored for summer-specific heat stress. |
| General Climate & UHI Mapping | Touati et al., 2020; Isinkaralar, 2024 | Surface temperature maps do not always correspond to direct human thermal discomfort. | Translates raw climate data directly into human bioclimatic stress categories (SSI). |
| SSI Calculations | Pepi, 1987; Adıgüzel & Doğan, 2021 | Previously limited to manual computations or point-based, small-scale areas. | Enables fast, reproducible spatial analysis at the district and city level. |
